# Application of a cognitive-structure framework-based scenario-based education programme in schizophrenia patients with delusional symptoms: a randomised controlled trial

**DOI:** 10.3389/fpsyt.2026.1822927

**Published:** 2026-05-28

**Authors:** Runing Hou, Yue Zhou, Kun Su, Xiaoyan Zhi, Fang Yan, Yanping Zhang, Lingfang Zhang

**Affiliations:** The Second Affiliated Hospital of Henan Medical University, Xinxiang, China

**Keywords:** cognitive biases, cognitive structural framework, delusions, schizophrenia, situational education

## Abstract

**Purpose:**

Antipsychotic medications have limited efficacy in improving cognitive function and social rehabilitation in patients with schizophrenia. Based on Beck’s cognitive theory, this study developed a structured educational programme and evaluated its short-term intervention effects on patients with schizophrenia presenting with delusional symptoms.

**Methods:**

We selected 102 patients with schizophrenia presenting with delusional symptoms from our hospital between January and December 2024, and randomly divided them into an observation group and a control group, each comprising 51 patients. The control group received standard pharmacological treatment and psychiatric care, whilst the observation group underwent an additional four weeks of scenario-based educational training based on the cognitive structure framework. Assessment was conducted using the SCSQ, C-CDRS, DACOBS and ERP-P300.

**Results:**

Forty-seven patients in the observation group and 45 in the control group completed the training. In the observation group, the SCSQ score for the positive coping factor was higher than that of the control group, whilst the score for the negative coping factor was significantly lower (P < 0.05); the total C-CDRS score and scores for each subscale, the total DACOBS score, and scores for safety behaviour and hasty conclusions were all superior to those of the control group (P < 0.05); the latencies of N1, N2 and P3 were shortened, and the amplitudes of N1 and P3 were increased (P < 0.05).

**Conclusion:**

Scenario-based education based on the cognitive structure framework can selectively improve patients’ coping strategies, delusional symptoms and certain cognitive distortions in the short term, accompanied by changes in neurophysiological indicators. However, the link between these changes and improvements in clinical cognitive function remains unclear. This study is a preliminary exploration; as it lacks a control group with matched characteristics and long-term follow-up, its conclusions are exploratory in nature.

## 1 Introduction

Schizophrenia is a chronic, progressive and highly disabling mental disorder, whose primary clinical manifestations involve disturbances in perception, thought processes and affect (1). Epidemiological data indicate that from 1990 to 2019, the global prevalence, incidence, and disability rates of schizophrenia have all risen significantly. Its lifetime prevalence remains approximately 0.7%–1.0%, and it continues to rank amongst the top ten causes of global health burden (2–5).

Among the various symptoms of schizophrenia, cognitive impairment has evolved from a secondary symptom to a core characteristic distinct from both positive and negative symptoms. The 2022 guidelines from the European Psychiatric Association indicate that at least 80% of patients exhibit varying degrees of cognitive impairment. This impairment does not automatically resolve with the alleviation of psychotic symptoms but rather constitutes a key factor constraining both disease outcomes and the restoration of social functioning in patients (6–8). At the same time, delusions, as one of the most common positive symptoms of schizophrenia, are often closely associated with specific cognitive distortions in their formation and maintenance. These distortions not only form the cognitive basis of delusional content but also constitute deep-seated barriers impeding patients’ reality testing abilities and motivation for recovery (9–11).

**Figure 1:**
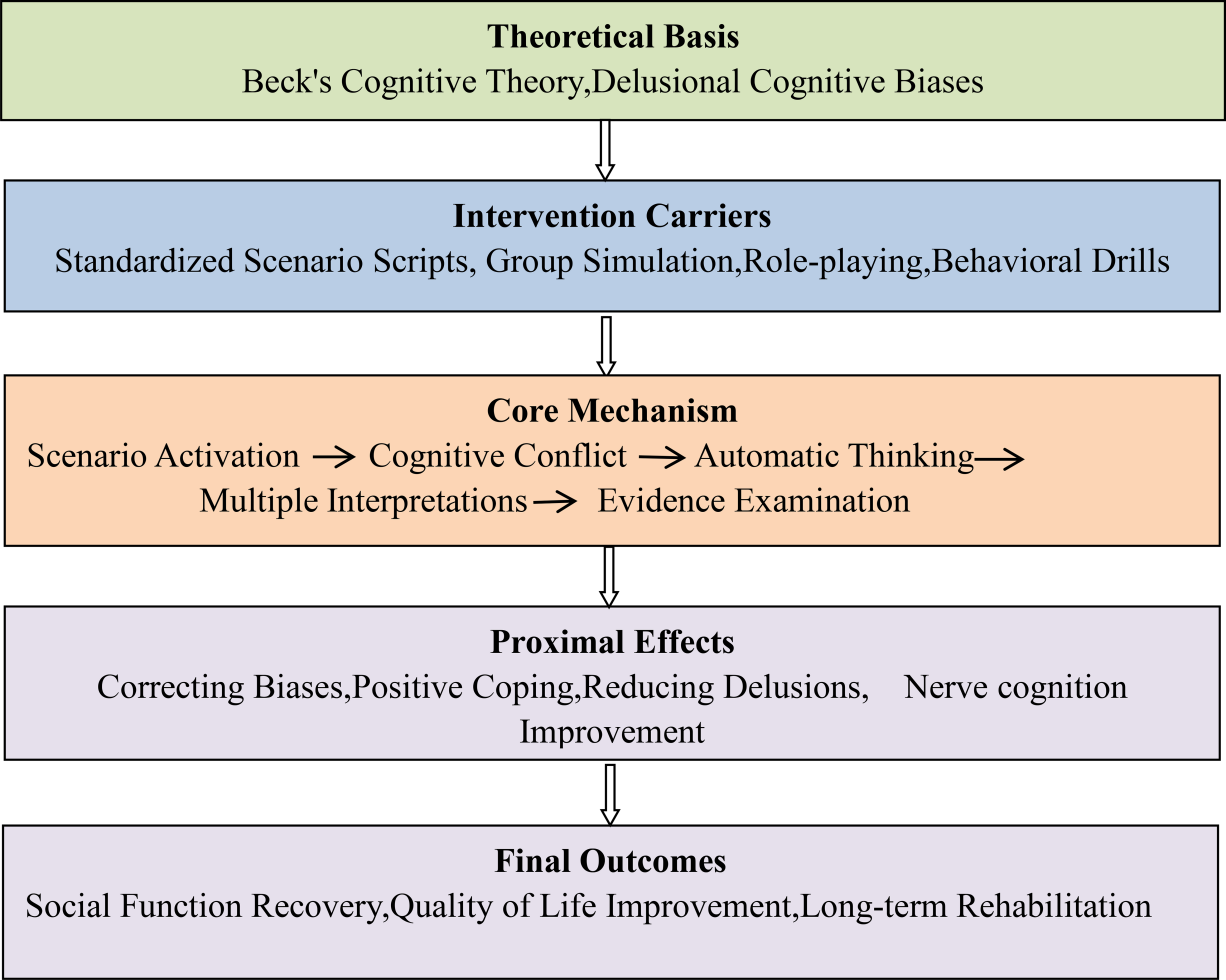
Schematic diagram of the cognitive structure intervention framework.

Presently, the treatment of schizophrenia remains predominantly reliant on antipsychotic medications. Whilst these drugs effectively alleviate patients’ psychotic symptoms, cognitive deficits persist in the majority of individuals following improvement of positive symptoms. Furthermore, some patients exhibit suboptimal responses to long-term treatment, resulting in limited enhancement of their quality of life and social functioning (12, 13). Therefore, combining psychosocial interventions with pharmacological treatment proves particularly crucial. Evidence-based research indicates that cognitive remediation therapy and cognitive behavioural therapy yield positive effects in improving patients’ cognitive function, coping strategies, and clinical symptoms (14–16). However, traditional cognitive behavioural therapy for delusions (CBT-p) relies primarily on verbal Socratic dialogue and an individual therapy model. Not only does this place high demands on the therapist’s professional expertise, but it is also constrained by factors such as significant individual differences and limited appeal to patients with severely impaired cognitive function, resulting in certain limitations in its clinical application and dissemination (17). Metacognitive training (MCT), on the other hand, specifically targets particular cognitive biases and enhances patients’ metacognitive awareness by correcting interpretative biases (18). It corrects cognitive biases through exercises and explanations, but lacks contextual experience and behavioural training. Scenario-based education, as an intervention paradigm that achieves the unity of knowledge, belief and action, activates learners’ cognitive restructuring and behavioural acquisition through simulated real-life scenarios, demonstrating unique potential in the field of mental rehabilitation (19). The ‘cognitive structural framework’ employed in this study is based on Beck’s cognitive theory (20). Designed to address the core cognitive distortions underlying delusions, it is a standardised group intervention framework comprising five levels: theoretical basis, intervention vehicle, core mechanisms, proximate effects, and ultimate outcomes (refer to **Figure 1**). Compared with CBT-p, this framework employs standardised group scenarios with a fixed protocol and low therapist dependency; compared with MCT, it places greater emphasis on experiential learning and behavioural rehearsal, which facilitates transfer to everyday life. Although the potential of scenario-based training interventions has received preliminary recognition, research integrating them closely with cognitive structural frameworks and systematically designing and empirically testing curricula targeting core cognitive distortions associated with delusions remains scarce. Most studies rely solely on subjective scales and lack objective neurophysiological indicators such as event-related potential P300 to corroborate the efficacy of interventions.

In summary, drawing on Beck’s cognitive theory, this study has developed a comprehensive intervention programme targeting the core cognitive distortions associated with delusional symptoms in schizophrenia. The programme integrates standardised scenario scripts, group role-play exercises, the induction of cognitive dissonance, and post-session journaling for consolidation. The study employs a multidimensional assessment system combining subjective and objective measures to systematically examine the impact of this intervention model on patients’ coping strategies, delusional beliefs, cognitive distortions and neurocognitive efficiency. Compared with existing intervention programmes, the core value of this study lies in its breakthrough in clinical implementation: within the resource-constrained setting of a hospital ward, it has established a standardised, replicable, and therapist-independent group intervention programme. This addresses the limitations of traditional CBT-p—which requires high levels of professional expertise—and MCT—which lacks experiential elements—thereby providing a new, low-cost and scalable psychosocial intervention pathway for the rehabilitation of schizophrenia.

## 2 Subjects and methods

### 2.1 Study subjects

**Figure 2:**
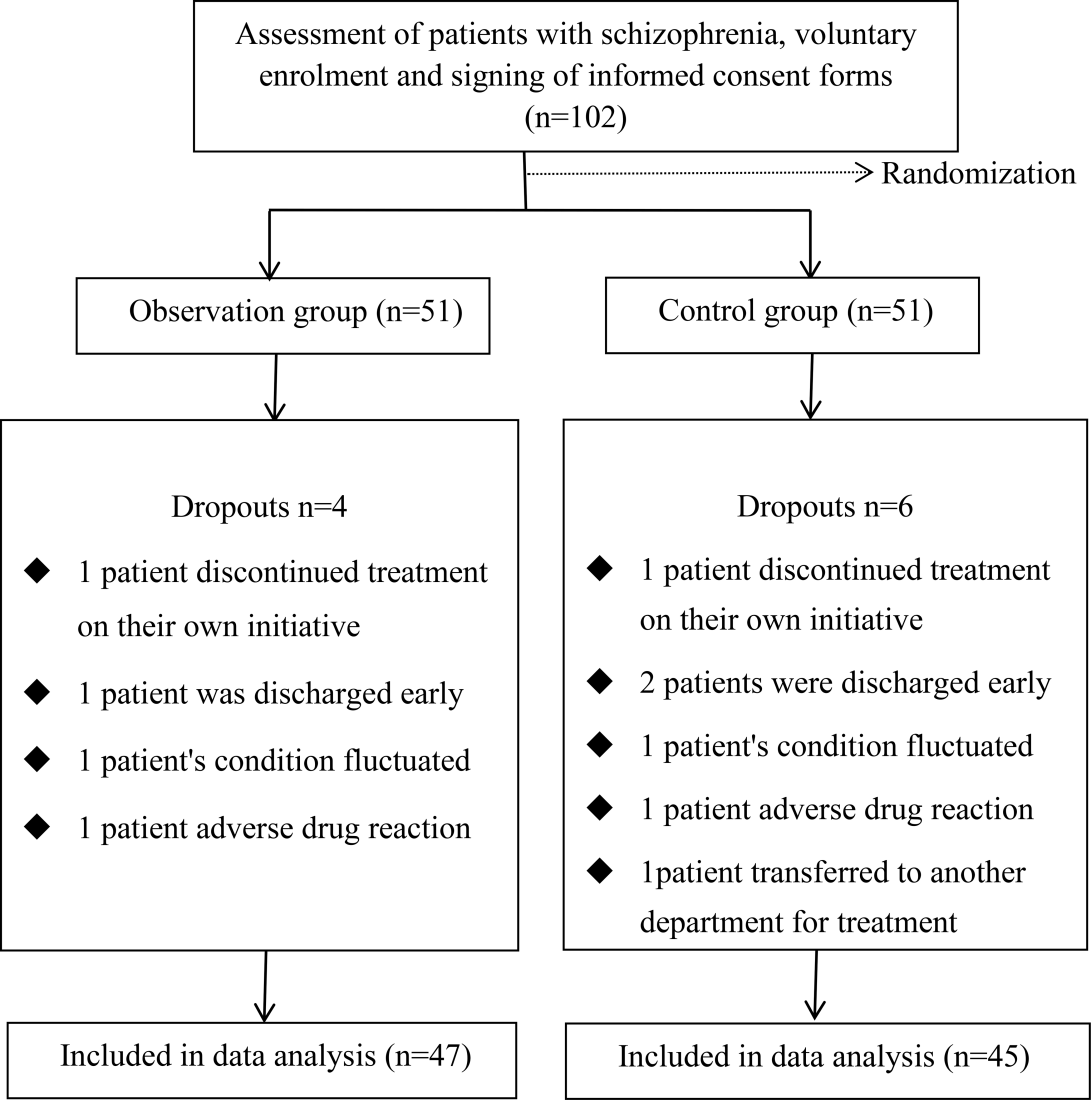
Flowchart of the study.

This study employed G-Power 3.1.9.7 software for sample size estimation, setting the effect size at 0.61,This is based on a meta-analysis of metacognitive training by Penney et al. (effect size of 0.69 for delusions) and an analysis by Mehl et al. of studies on causal intervention-oriented CBTp (effect size of 0.60) (21, 22), test power (1-β) at 0.80, and dividing the sample into two groups. With a significance level (α) of 0.05 using a two-tailed test, calculations determined that each group required a sample size of 43 subjects. Based on a previous study by the research team involving a similar population (in which the dropout rate was approximately 9%) (23), and to account for uncertainty regarding hospitalised patients, a dropout rate of 15% was assumed; accordingly, it was calculated that 51 patients would need to be enrolled in each group. The study initially enrolled 102 participants, thereby meeting the statistical requirements. The study population comprised 102 patients with schizophrenia admitted to the Second Affiliated Hospital of Henan Medical University between January and December 2024. During the study period, 10 patients were lost to follow-up due to reasons including voluntary discontinuation, early hospital discharge, disease fluctuations, and adverse drug reactions. This comprised 4 cases in the observation group and 6 in the control group. A total of 92 patients ultimately completed the study, with 47 in the observation group and 45 in the control group completing the entire research protocol. All meet the minimum sample size requirements. This study did not employ an intention-to-treat (ITT) analysis, but instead used an in-protocol analysis; this may have resulted in a slight overestimation of the intervention effect size. The specific workflow of this study is illustrated in **Figure 2**.

Inclusion criteria: (1)Meets the diagnostic criteria for schizophrenia as outlined in the International Classification of Diseases, 10th Revision (ICD-10), with the presence of delusional symptoms; (2)The type and dosage of pre-enrolment medication have remained stable for over two weeks, with no modifications to the treatment regimen, and psychiatric symptoms are relatively stable; (3)Positive and Negative Symptom Scale (PANSS) score (24) ≥ 70 points, with delusional symptoms scoring > 3 points on the PANSS items; (4)At least secondary school level education with no impediments to verbal communication, and able to maintain concentration for the majority of the time.

Exclusion criteria: (1) Individuals with substance abuse disorders; those with severe physical illnesses and organic brain disorders; (2) Individuals with severe impulsivity or suicidal ideation; those undergoing psychotherapy within other systems. This study has been reviewed and approved by the Ethics Committee of the Second Affiliated Hospital of Henan Medical University, with approval number XYEFYLL-(Scientific Research)-2024-95.

### 2.2 Classifications and blindness

In this study, patients were randomised using a random number table. An independent statistician, who was not involved in the clinical intervention or the assessment of outcomes, selected two random digits in sequence from the table, starting from any point, and assigned these to each patient in the order of enrolment. It was stipulated that participants with odd-numbered randomised codes would be allocated to the observation group, and those with even-numbered codes to the control group, with 51 participants to be included in each group. If one group reaches its target sample size early, subsequent patients will be automatically allocated to the other group, resulting in 51 patients in each group. Once the random sequence has been generated, the allocation results are placed in sequentially numbered, sealed, opaque envelopes. The investigator opens the envelopes in the order of patient enrolment to determine the group allocation.

Due to the nature of the intervention (scenario-based education requires interaction between the therapist and the patient), it is not possible to conduct a double-blind study involving therapists and patients. However, none of the outcome assessors (including those administering the scales and conducting the ERP-P300 testing) were aware of the group allocation. Data analysis was carried out by statisticians who were not involved in the implementation of the intervention, and unblinding took place after data lock-in. Once data entry is complete, the project manager will verify the data; once it has been confirmed to be accurate, the database will be locked, and the statisticians will then carry out the final analysis.

### 2.3 Research methods

#### 2.3.1 Establish the intervention team

The intervention team comprises two psychiatrists, one senior psychiatric nurse practitioner, two deputy senior psychiatric nurse practitioners, two specialist mental health nurses (all holding National Level II Psychological Counsellor qualifications), and six psychiatric nurses. Among these, the psychiatrist and the Deputy Head Nurse are responsible for subject selection and script review; The Head Nurse of the Psychiatric Department is responsible for developing the curriculum framework. Mental health specialist nurses are responsible for drafting scenario-based event scripts, implementing training programmes, and collecting, collating, and statistically analysing data; Psychiatric nurses then assume their roles within the script to facilitate the training. Prior to the commencement of training, all members of the training team shall undergo standardised training on relevant knowledge and the training programme.

#### 2.3.2 Standard development and finalisation of scenario scripts

Building on this research group’s previous scenario-based training programme for patients with schizophrenia (23), the content is designed around common delusional symptoms encountered in psychiatric clinical practice—such as delusions of reference, persecutory delusions, delusions of non-blood relationship, and delusions of jealousy—as triggering events, with scenarios developed in the context of daily life and interpersonal interactions. To ensure standardisation, each scenario includes the following elements: (1) a fixed narrative framework (setting, characters, triggering event); (2) 3–4 predefined key decision points (cognitive biases often manifest at these junctures); (3) standardised prompts for each decision point; (4) multiple outcomes illustrating different responses.

The development process was divided into three phases. In the first phase (script drafting), two psychiatric specialist nurses drafted the script based on their clinical experience and the literature on cognitive biases; in the second phase (patient pilot testing), 10 patients with schizophrenia who had delusions but were in a stable condition (and who did not participate in the subsequent formal trial) were invited to take part in two pilot sessions, each lasting 1–1.5 hours. Feedback from patients regarding the script’s comprehensibility, emotional relevance and realism was recorded; Stage 3 (Expert Review): Four external experts (two psychiatrists and two cognitive behavioural therapists) independently reviewed the script using a standardised checklist (content validity, targeting of cognitive distortions, and feasibility). Consensus was reached following three rounds of revisions. The final curriculum comprises seven themes, each containing two scenarios (with the exception of Themes 1 and 7), as shown in **Table 1**.

**Table 1:** Situational education curriculum based on cognitive structure framework.

Course module	Theme	Core content	Implementation steps
Foundation-Building Phase (Session 1)	Establishing Collaborative Relationships	Icebreaker Activities + Cognitive Introduction	1. Icebreaker games (e.g., “Name Chain”) to foster a relaxed atmosphere; 2. Patient self-introductions, sharing perceptions of their own “abnormal thoughts”; 3. PowerPoint presentation explaining the nature and impact of delusions, introducing training objectives, rules, and log-keeping methods.
Cognitive Awakening Phase (Sessions 2–5)	Multi-dimensional Cognitive Training	Challenging the notion of a “single correct answer” Cognitive	1. Scenario Demonstration: Present the scenario where “colleagues cease conversation upon seeing you enter”. Observe how this event—two colleagues engaged in lively conversation halting their chat when you walk into the office—yields differing outcomes based on cognitive patterns, such as confronting them directly, harbouring silent resentment, indifference, or joining the conversation. 2. Group Discussion: Participants share initial reactions (e.g., “They’re gossiping about me”, “They’d just finished talking”).3. Guided Reflection: Expand cognitive dimensions through prompts like “Are there other possibilities?” and “How would you interpret this situation?”
	Evidence Gathering Exercise	Learning to make rational judgements based on evidence	1. Scenario demonstration: “Discovering a needle on a pillow.” First, allow patients to express thoughts like “being framed” and their reasoning; 2. Revealing the truth: Demonstrate the complete process showing “the needle was left behind during pillow mending”; 3. Practical exercise: Patients share past incidents where they “judged based on intuition,” with the group assisting in identifying “collectable evidence.”
Cognitive Restructuring Phase (Sessions 6–9)	Irrationality Dispelling Irrational Beliefs	Cognitive Bias Correction	1. Scenario Replay: Present the “Xiaoyue pushing someone” incident in four segments (initially showing only the “pushing” outcome → adding “provocation by the pushed individual” → supplementing with “Xiaoyue’s failed attempts to dissuade” → complete cause-and-effect sequence); 2. Stage Reflection: After each segment, prompt the patient to analyse causes and compare judgment differences across stages; 3. Method Instruction: Teach rational thinking techniques such as “empathy” and “holistic observation”.
	Transforming Fixed Mindsets	Understanding the Impact of Thought on Behaviour	1. Scenario demonstration: “Du Xiao lashes out at parents during visit, claiming they are not his biological parents”; 2. Socratic questioning: Guide the patient to consider “What specific acts of care have parents shown?” “Beyond ‘acting’, are there other explanations?”; 3. Alternative thinking exercise: The patient writes down their entrenched beliefs and attempts to list at least three alternative interpretations.
Behavioural Consolidation Phase (Sessions 10-11)	Selecting Coping Strategies	Distinguishing Positive from Negative Coping	1. Scenario Comparison: Demonstrate two responses to “wife discovering husband alone with female colleague late at night” (positive: communication and inquiry; negative: suspicion and blame) and their consequences; 2. Case Sharing: Patients recount coping experiences related to delusions; group discussion to refine strategies; 3. Role-Play: Patients swap roles to practise positive coping approaches.
Consolidation and Reinforcement Phase (Session 12)	Sharing and Summary	Consolidating Training Outcomes	1. Standardised patient demonstration: Presenting the complete process of “managing delusion-related incidents following cognitive restructuring”; 2. Patient sharing: Discussing changes in thoughts, emotions, and behaviours before and after training, presenting training logs; 3. Recognition of achievements: Commending actively participating patients, summarising core techniques, and encouraging continued application of training methods in daily life.

“Colleagues stopping their conversation” targets egocentric attribution bias and selective attention to threats, corresponding to biases associated with delusions of reference; “A needle in the pillow” targets hasty generalisation and insufficient evidence collection, corresponding to biases associated with persecutory delusions; “Xiaoyue pushing someone” targets one-sided attribution, hostile attribution and jumping to conclusions, corresponding to social cognitive impairment; “Du Xiao claiming the child is not his own” targets fixed beliefs and insufficient mentalisation, corresponding to delusions of non-paternity; “Wife seeing husband alone with a female colleague” targets safety behaviours and selective attention to threats, corresponding to delusions of jealousy.

Each scenario, through built-in ‘cognitive conflict’ triggers, specifically targets one or two predefined cognitive biases. For example, the ‘needle found in a pillow’ scenario addresses ‘jumping to conclusions’; by revealing information in stages (first presenting the outcome, then providing the full explanation), the scenario directly challenges the bias of hasty conclusions. The ‘colleague stops talking’ scenario presents multiple possible explanations, targeting egocentric attribution and the threat to selective attention. The “Family Visiting Conflict” scenario requires patients to list at least three alternative explanations, targeting belief rigidity and insufficient mentalisation. At the conclusion of each scenario, the therapist facilitates a group discussion to explicitly identify the targeted biases and their manifestations in real life, thereby promoting the transfer of learning.

#### 2.3.3 Training programme

Control group: received standard medical treatment and psychiatric care.

Observation group: In addition to the control group, this group received training in a scenario-based educational programme based on a cognitive structural framework; this framework is grounded in Beck’s cognitive theory and designed to address cognitive distortions associated with delusions. As shown in **Figure 1**, this cognitive structural framework is divided into five levels: theoretical basis, intervention vehicle, core mechanism, proximate effect and final outcome, clearly illustrating the complete intervention pathway from situational activation to cognitive restructuring.

Each group comprised 8 to 12 patients, with sessions conducted three times weekly for 1.0 to 1.5 hours per session, over a four-week training cycle. The training programme is divided into five stages, comprising seven themes: Theme 1 ‘Establishing Collaborative Relationships’ and Theme 7 ‘Sharing and Summarising’ each comprise one session; the remaining five themes each unfold through two scenario-based events, each requiring two sessions to complete. Prior to training, it is essential to gain a thorough understanding of the patient’s social background, personality traits, and the content of their delusions, thereby completing the data collection and assessment process. Following each scenario-based training session, assign homework to guide patients in integrating the training methods into their daily lives; to enhance patient adherence to the training programme, specially designed training log sheets were created. These sheets are tailored to the training content, are simple and easy to understand, and are convenient to complete. Patients were also instructed on how to fill them in. The table covers six aspects: (1)What life event are you currently experiencing? (2)When an incident occurs, endeavour to identify multiple cognitive-level explanations for the event. (3)What constitutes the basis for determining the various cognitive causes? (4)Which cognitive cause ultimately prevails? (5)What measures have been taken in response? (6)What were the outcomes (including emotional changes, psychological experiences, impact on daily life, etc.)? Through this design, patients are guided to progressively learn alternative thought patterns within the “event-thought-coping strategy-behavioural consequence” model. This facilitates cognitive restructuring, enables mastery of positive coping mechanisms, and enhances their capacity to address challenges effectively. Before each training session, select two to three patients to take turns sharing their journals; a monetary reward will be given at the end of the course. All group members must complete the full training programme within four weeks. The course schedule is detailed in **Table 1**.

Both groups of patients received small gifts of equal value (such as toilet paper, toothbrushes, soap and other everyday essentials).Patients in the control group were given the questionnaire after completing the assessment, whilst those in the observation group were given it after completing each session. The two sets of small gifts are of equal value, and the rewards are not directly linked to the primary outcome measures. All outcome measures were assessed by evaluators who were unaware of the group allocation, and objective neurophysiological measures were included; consequently, it is unlikely that this incentive scheme would introduce substantial bias in the comparison between groups.

### 2.4 Evaluation metrics.

#### 2.4.1 Coping style

Measurements were taken using the Simplified Coping Style Questionnaire (SCSQ) before and after the training session. This questionnaire developed by Xie Yaning et al. assesses patients’ attitudes and measures adopted when encountering difficulties in daily life. The scale comprises two dimensions—positive coping and negative coping—comprising 20 items scored on a four-point scale ranging from 0 to 3.Positive coping comprises items 1 to 12, with a total score ranging from 0 to 36 points; negative coping comprises items 13 to 20, with a total score ranging from 0 to 24 points. Higher scores indicate better positive coping, whilst lower scores indicate better negative coping. Research findings indicate that the internal consistency coefficients for positive coping and negative coping within this scale were 0.89 and 0.78 respectively, whilst the overall scale demonstrated an internal consistency coefficient of 0.90, exhibiting sound reliability and validity (25).

#### 2.4.2 Severity of delusional symptoms

Measurements were taken using the Chinese Version of the Delusional Characteristics Rating Scale (C-CDRS). The C-CDRS was administered pre- and post-intervention. This scale comprises three factors: cognitive, affective, and oddity, encompassing eleven items: degree of conviction, degree of preoccupation, degree of interference, degree of resistance, degree of disintegration, degree of absurdity, degree of self-evidence, degree of seeking confirmation from others, degree of causing distress, degree of causing unpleasantness, and degree of diffusion of beliefs. The cognitive factor comprises items 1, 2, 3, 5, 8, and 11; the affective factor comprises items 4, 9, and 10; the strangeness factor comprises items 6 and 7.Each item is rated on a 10-point scale from 1 to 10, where 1 denotes the lowest severity and 10 denotes the highest severity. The total score is the sum of the scores for all 11 items; a higher total score indicates more severe delusional symptoms in the patient. The literature indicates that the Cronbach’s α coefficient for the total C-CDRS score is 0.83, with the three factor-specific coefficients being 0.84, 0.77, and 0.78 respectively. The scale demonstrates good reliability and validity, rendering it suitable for use in the assessment of delusions amongst patients with schizophrenia (26).

#### 2.4.3 Cognitive bias

Measurements were taken using the Chinese Version of the Davos Cognitive Bias Assessment Scale (DACOBS). The Yang-Jin-Yu Revised DACOBS was employed for pre- and post-intervention assessment. This scale comprises seven factors: cognitive subjectivity, social cognitive impairment, insufficient mentalisation, safety behaviours, hasty conclusions, belief rigidity bias, and selective attention to threats, comprising 36 items (with items 1, 3, 4, 5, 12, and 22 from the original version omitted).The cognitive subjectivity factor comprises items 21, 28, 32, 33, 36, and 40; the social cognitive impairment factor comprises items 6, 11, 23, 24, 39, and 42; the metacognitive deficiency factor comprises items 2, 14, 15, 17, 19, and 29; the safety behaviour factor comprises items 27, 31, 34, 35, 42; the hasty judgement factor comprises items 8, 16, 18, 25, 30, 38; the belief rigidity bias comprises items 7, 9, 13, 26; and the selective attention to threats factor comprises items 8, 10, 20, 37, 41.Each entry is stated in the first person, reflecting attitudes or beliefs characteristic of human beings. Scores range from 1 to 7, where ‘7’ indicates strong agreement and ‘1’ indicates strong disagreement, with decreasing intensity thereafter. Entries 8 and 42 are double-scored. The higher the total score, the greater the cognitive bias. The literature indicates that the Cronbach’s α coefficient for the total DACOBS score is 0.92, with Cronbach’s α coefficients for all seven factors ranging between 0.66 and 0.81. These values are comparable to those of the English version (0.64–0.82). The scale demonstrates good reliability and validity for assessing cognitive distortions in schizophrenia (27).

#### 2.4.4 The brain’s attention, information processing speed, cognitive judgment, and working memory abilities

Measurements were taken using the Event-Related Potential P300 (ERP-P300). The MEB-9404C electromyography-evoked potential recorder manufactured by NIHON KOHDEN Corporation of Japan was employed for ERP-P300 measurement. The laboratory requires acoustic shielding, a quiet environment, and suitable temperature and humidity conditions. Following the international 10/20 system, recording electrodes are placed at the subject’s Cz and Fz positions, with reference electrodes positioned at the subject’s left or right earlobe. An Oddball auditory stimulus pattern is employed, with non-target stimuli occurring at an 80% probability, at an intensity of 70 dB, with a frequency of 1,000 Hz. Target stimuli occur with a 20% probability at 90 dB intensity and 2,000 Hz frequency. Both types appear randomly. Subjects respond to target stimuli by pressing a button. Electrode impedance is maintained below 5 kΩ, with filtering applied between 0.5 and 100.0 Hz. Analysis time is set to 500 ms, measuring the latency and amplitude of N1, N2, and P3 waves. A longer latency period indicates slower information processing speed in patients. A lower amplitude signifies poorer attention, cognitive judgement, and working memory capacity in the patient’s brain.

### 2.5 Statistical methods

Statistical analysis was performed using SPSS 27.0 software. Quantitative data are presented as mean ± standard deviation ( $\bar{x}$ ± s) and analysed using the independent samples t-test; qualitative data were assessed using the χ² test. P value < 0.05 was considered statistically significant. As this study included only a conventional treatment and care control group and did not include an equal-dose attention-matched control group, it was unable to fully distinguish between the specific effects of the intervention and non-specific effects such as group contact and the level of therapeutic attention. Furthermore, as an intention-to-treat (ITT) analysis was not conducted, the results of this study may be subject to a bias that overestimates the effect of the intervention; therefore, the findings should be interpreted with caution.

### 2.6 Quality control and intervention fidelity

(1)Before the study began, the two psychiatric nurses received standardised training in psychological assessment, familiarised themselves with the questionnaire content and unified the guidance, to ensure consistency when patients completed the questionnaire. (2)The ERP-P300 measurement is carried out by experienced professional technicians. (3)Appoint a senior expert to oversee the collection of information and promptly identify and resolve any issues. (4)In the early, mid-term and late stages, a random selection of the intervention process is recorded, with the recordings then being reviewed by a senior therapist who has not been involved in the intervention, who assesses whether the intervention is being carried out as planned and provides feedback. (5)A weekly training session is held for the rehabilitation therapists implementing the intervention, to ensure consistency of implementation and adherence to key procedures. Any deviations from the plan are discussed and corrected during the training session. (6)All data is checked against the original records. The team leader carries out regular checks to ensure the completeness and accuracy of the data. The initial draft was independently completed by two researchers with CET-6 (College English Test Band 6) proficiency. Subsequently, they conducted a cross-exchange review, focusing on the grammatical accuracy and terminology consistency of the translation. Finally, the project leader performed the final review to ensure the professionalism and linguistic authenticity of the translated text.

## 3 Results

### 3.1 Comparison of baseline characteristics between the two patient groups

This study enrolled a total of 102 patients. Using a randomised number table, they were divided into an observation group and a control group, each comprising 51 patients. During the study, 10 patients were lost to follow-up due to reasons including voluntary discontinuation, early discharge, disease fluctuations, and adverse drug reactions. Of these, 4 were from the observation group and 6 from the control group. Ultimately, 92 patients completed the study, resulting in 47 patients in the observation group and 45 in the control group. There were no statistically significant differences between the two patient groups in terms of gender, age, educational attainment, marital status, duration of illness, type of delusion, or chlorpromazine-equivalent dose (P > 0.05), as shown in **Table 2**.

**Table 2:** Comparison of baseline characteristics between the two patient groups [cases, percentage (%)].

Item	Category	Observation group (n=47)	Control group (n=45)	Statistical measure	P-value
Gender	Male	23 (48.9)	21 (46.7)	0.047^1^	0.828
	Female	24 (51.1)	24 (53.3)		
Age		34.70 ± 9.88	34.64 ± 6.31	0.034^2)^	0.974
Level of education	Junior secondary	20 (42.6)	13 (28.9)	4.019^1^	0.134
	Senior Secondary	10 (21.3)	18 (40.0)		
	College and above	17 (36.2)	14 (31.1)		
Marital status	Unmarried	18 (38.3)	18 (40.0)	1.777^1^	0.411
	Married	25 (53.2)	26 (57.8)		
	Divorced/Widowed	4 (8.5)	1 (2.2)		
Duration of illness (years)	<2	7 (14.9)	6 (13.3)	0.150^1^	0.928
	2–10	30 (63.8)	28 (62.2)		
	>10	10 (21.3)	11 (24.4)		
Type of delusion	Relationship delusions	15 (31.9)	20 (44.4)	2.392^1^	0.495
	Persecutory delusions	28 (59.6)	22 (48.9)		
	Jealousy delusions	3 (6.4)	3 (6.7)		
	Other types	1 (2.1)	0 (0.0)		
Chlorpromazine equivalent dose		289.36 ± 119.75	316.67 ± 75.38	-1.315^2)^	0.192

1) *χ*^2^ Value; 2) t-value.

### 3.2 Comparison of SCSQ scores before and after training in the two patient groups

Prior to training, comparisons of SCSQ scores for the positive coping dimension and negative coping dimension between the observation group and the control group revealed no statistically significant differences (P > 0.05). Following the training programme, both groups exhibited a significant increase in positive coping scores and a significant decrease in negative coping scores compared to pre-training levels (P < 0.05). Furthermore, the observation group demonstrated significantly higher positive coping scores and significantly lower negative coping scores than the control group, with both differences being statistically significant (P < 0.05), as shown in **Table 3**.

**Table 3:** Comparison of SCSQ scores before and after training in both patient groups ( $\bar{x}$ ± s, points).

Time	Group	Number of cases	Positive coping	Passive coping
Pre-training	Observation group	47	17.40 ± 3.26	13.66 ± 2.51
	Control group	45	18.20 ± 3.15	13.62 ± 2.69
	t-value		-1.189	0.069
	P value		0.238	0.945
Post-training	Observation group	47	24.51 ± 2.40 ^*^	8.34 ± 1.80 ^*^
	Control group	45	19.98 ± 3.52	11.60 ± 3.78
	t-value		7.184	-7.393
	P value		P < 0.001	P < 0.001

*Compared with pre-training levels in this group, *P* < 0.05.

### 3.3 Comparison of C-CDRS scores before and after training in the two patient groups

Prior to training, comparisons of C-CDRS subscale scores between the observation group and control group revealed no statistically significant differences (P > 0.05). Following training, both groups exhibited significantly reduced C-CDRS subscale scores compared to pre-training levels, with the observation group demonstrating markedly lower scores than the control group. These differences were statistically significant (P < 0.05), as shown in **Table 4**.

**Table 4:** Comparison of C-CDRS scores before and after training in two patient groups (x̄ ± s, points).

Time	Group	Number of cases	Cognitive factor	Emotional factor	Eccentricity factor	Total score
Pre-training	Observation Group	47	41.87 ± 3.52	17.32 ± 4.26	11.98 ± 1.31	71.17 ± 6.77
	Control Group	45	42.64 ± 2.34	18.11 ± 1.93	11.44 ± 1.44	72.20 ± 2.90
	t-value		-1.245	-1.157	1.863	-0.955
	P value		0.217	0.252	0.066	0.343
Post-training	Observation group	47	25.02 ± 3.11 ^*^	11.45 ± 2.21 ^*^	7.83 ± 1.68 ^*^	44.30 ± 3.67 ^*^
	Control group	45	32.38 ± 3.67	14.47 ± 1.75	8.82 ± 1.75	55.67 ± 4.92
	t-value		-10.388	-7.231	-2.772	-12.602
	P value		P < 0.001	P < 0.001	0.007	P < 0.001

*Compared with pre-training in this group, *P* < 0.05.

### 3.4 Comparison of DACOBS scores before and after training in the two patient groups

Prior to training, comparisons of total DACOBS scores and factor-specific ratings between the observation group and control group revealed no statistically significant differences (P > 0.05). Post-training, the observation group demonstrated a greater reduction in total DACOBS scores compared to the control group, with statistically significant differences observed (P < 0.05). Scores for safety behaviours and jumping to conclusions both decreased significantly compared to the control group, with statistically significant differences (P < 0.05). The reduction in scores for cognitive subjectivity, social cognitive deficits, metacognitive deficits, belief rigidity bias, and selective attention to threats was greater than that of the control group; however, no statistically significant differences were observed in the five-factor scores (P > 0.05), as shown in **Table 5**.

**Table 5:** Comparison of DACOBS scores before and after training between patient groups (x̄ ± s, points).

Factor	Group	Observation group (n=47)	Control group (n=45)	t-value	p-value
Cognitive Subjectivity Issues	Pre-training	19.64 ± 2.15	19.58 ± 2.34	1.290	0.897
	Post-training	17.68 ± 2.11	18.48 ± 2.37	-1.730	0.087
Social cognitive impairment	Pre-training	18.91 ± 4.50	19.36 ± 4.87	-0.451	0.654
	Post-training	16.34 ± 3.68	17.36 ± 4.70	-1.156	0.251
Insufficient mentalisation	Pre-training	18.91 ± 3.62	15.71 ± 2.58	-0.122	0.911
	Post-training	17.98 ± 3.70	18.16 ± 3.39	-0.239	0.812
Safety behaviour	Pre-training	15.55 ± 3.13	15.71 ± 3.24	-0.238	0.812
	Post-training	12.04 ± 3.18^*^	14.38 ± 3.37	-3.417	P < 0.001
Premature conclusions	Pre-training	22.94 ± 2.68	22.89 ± 3.23	0.077	0.939
	Post-training	18.57 ± 2.98^*^	20.82 ± 3.09	-3.553	P < 0.001
Belief rigidity bias	Pre-training	13.06 ± 2.56	13.33 ± 2.74	-0.487	0.627
	Post-training	12.17 ± 2.48	13.17± 2.68	-1.869	0.065
Selective attention to threats	Pre-training	19.81 ± 2.76	20.11 ± 2.81	-0.520	0.604
	Post-training	18.78 ± 2.75	19.82 ± 2.88	-1.764	0.081
Total Score	Pre-training	128.85 ± 11.49	130.00 ± 9.31	-0.526	0.600
	Post-training	113.57 ± 10.99^*^	122.20 ± 8.90	-4.119	P < 0.001

*Compared with pre-training values in this group, *P* < 0.05.

### 3.5 Comparison of ERP-P300 parameters before and after training in the two patient groups

Prior to training, comparisons of ERP-P300 metrics between the two patient groups revealed no statistically significant differences (P>0.05). Following training, the observation group exhibited shorter latencies for N1, N2, and P3 components compared to the control group, alongside higher N1 and P3 amplitudes, with statistically significant differences (P<0.05). However, no statistically significant difference was observed between groups for N2 amplitude (P>0.05), as shown in **Table 6**.

**Table 6:** Comparison of ERP-P300 parameters between groups before and after training (x̄ ± s, points).

Time	Group	Number of cases	Latency (ms)			Amplitude (μV)		
			N1	N2	P3	N1	N2	P3
Pre-training	Observation group		115.00 ± 10.14	244.25 ± 20.36	339.11 ± 20.89	6.59 ± 1.23	5.19 ± 1.33	7.51 ± 1.91
	Control group	47	119.04 ± 9.96	247.16 ± 21.37	340.78 ± 21.99	6.22 ± 1.59	5.16 ± 1.28	7.51 ± 1.94
	t-value	45	-1.929	-0.666	-0.374	1.263	0.132	-0.001
	P value		0.057	0.507	0.709	0.210	0.895	0.999
Post-training	Observation group		98.89 ± 8.47 ^*^	226.95 ± 20.44^*^	315.91 ± 18.65^*^	8.89 ± 1.32^*^	6.19 ± 1.33 ^*^	10.11 ± 1.72 ^*^
	Control group	47	104.71 ± 9.28	237.75 ± 20.94	326.58 ± 21.94	8.04 ± 1.24	5.75 ± 1.29	9.13 ± 1.63
	t-value	45	-3.144	-2.503	-2.515	3.171	1.590	2.779
	P value		0.002	0.014	0.014	0.002	0.115	0.007

*Compared with pre-training values in this grop, *P* < 0.05.

## 4 Discussion

### 4.1 Reshaping response strategies: from passive avoidance to active engagement

The results of this study show that patients in the observation group demonstrated greater improvement than those in the control group on both the positive coping and negative coping dimensions, with statistically significant differences (*P* < 0.05). This aligns with the majority of current research findings. Analysis suggests that scenario training may assist patients in adopting more positive coping strategies when managing life events, thereby reducing negative coping behaviours and mitigating the adverse effects of delusional symptoms. This study’s scenario training is grounded in a cognitive structural framework, enabling patients to directly experience the differing emotional and relational consequences arising from various coping strategies; Through group interaction and role-play, patients are provided with opportunities to practise and reinforce positive coping skills such as expressive communication, evidence-seeking, and perspective-taking. This approach concretely demonstrates positive coping strategies and their effects, enabling patients to personally experience the superior behavioural outcomes resulting from shifts in thought patterns and coping mechanisms; Simultaneously, patients gain positive emotional experiences by witnessing the affirmation exchanged between the therapist and group members, thereby breaking the vicious cycle of negative coping, adverse outcomes, and reinforced delusions. Moreover, the diversity of thought patterns and shared experiences amongst patients within the group constitutes a multifaceted source of influence, providing rich resources and contextual references for rehabilitation training. Through group interaction, patients observe and learn from other members’ constructive coping strategies. By accumulating experience and enhancing problem-solving abilities, they gradually rebuild self-confidence and self-esteem, thereby developing more effective positive coping strategies. This finding is also consistent with the conclusions of numerous studies: A randomised controlled trial conducted by Katsushima et al. (28) examining the effects of video-conference-based cognitive behavioural therapy on patients with schizophrenia found that this approach demonstrated significant efficacy in reducing positive symptoms, alleviating depression, enhancing coping skills, and improving illness awareness. This aligns with the findings of Taha et al. (29), which indicate that cognitive behavioural therapy can effectively reduce the severity of delusions and hallucinations in individuals with schizophrenia whilst enhancing their coping abilities. Glanaghy et al. (30) analysed 90 randomised controlled trials (involving 8,440 participants) comparing the efficacy of 24 psychological interventions against control methods. The results demonstrated that psychological interventions were generally superior to control groups, with mindfulness-based cognitive behavioural therapy proving most effective in alleviating overall symptoms. A proactive coping approach assists patients in better managing life’s stresses and adverse events, thereby enhancing psychological resilience. This aligns with previous research findings on cognitive behavioural therapy for schizophrenia patients, which demonstrated that cognitive restructuring and behavioural training can effectively improve patients’ coping abilities.

### 4.2 Alleviation of delusional symptoms: shaking the conviction of belief and emotional investment

The findings of this study indicate that the total C-CDRS score and the scores for the cognitive, affective, and oddness subscales in the observation group decreased significantly, with reductions greater than those in the control group. These differences were statistically significant (*P* < 0.05). Analysis indicates that the reason may lie in the fact that this study employed cognitive structural scenario training to implement effective interventions targeting the formation mechanisms of delusions. This approach not only alleviated the delusional beliefs themselves but also reduced the associated emotional distress and bizarre attributes. Delusions manifest as fixed beliefs lacking factual basis; for instance, individuals with persecutory delusions may firmly believe they are under threat or being persecuted. Despite such beliefs being manifestly at odds with reality, they often prove stubborn and resistant to change. Schizophrenic patients exhibiting delusional symptoms tend to focus more intensely on environmental factors, attributing events to external causes. Moreover, the more severe the delusional symptoms, the more pronounced their personalised biases become. In the ‘evidence collection training,’ through the progressive disclosure of information in scenarios such as ‘the needle in the pillow,’ patients are guided to personally experience the process from ‘hasty conclusions’ to ‘discovering the truth.’ This essentially constitutes a form of behavioural experiment, effectively reducing confidence in initial erroneous beliefs. The results are comparable to those of Berkhof et al.’s cognitive behavioural therapy for virtual reality paranoia and other cognitive behavioural therapies (31). The “Multidimensional Thinking Training” module employs group discussions to deliberately expose patients to multiple plausible interpretations of the same event. This directly challenges the egocentricity and interpretative biases inherent in their cognition, thereby undermining their entrenched and peculiar personalised beliefs. The improvement in emotional factors may be a concomitant process: as patients begin to question the factual basis of their delusional beliefs, accompanying negative emotions such as anxiety, anger, and fear naturally subside. This subsequently reduces impulsive aggressive behaviour, aligning with the principle in cognitive behavioural therapy that ‘cognitive reappraisal guides emotional change’ (32). Shukla et al. investigated the efficacy and durability of cognitive behavioural therapy in managing hallucinations amongst patients with schizophrenia. Results indicated that, compared with medication alone, the combination of cognitive behavioural therapy and medication proved more effective in improving clinical symptoms and overall functioning in schizophrenia (33).

### 4.3 Correction of cognitive biases: interventions targeting core cognitive mechanisms

The findings of this study indicate that the reduction in total DACOBS scores was greater in the observation group than in the control group, with a statistically significant difference (*P* < 0.05). Scores for safety behaviours and rash judgements showed a significant decrease compared to the control group, with a statistically significant difference (*P* < 0.05).Moreover, participants demonstrated greater reductions than the control group in cognitive subjectivity issues, social cognitive deficits, metacognitive insufficiency, belief rigidity bias, and selective attention to threats; however, differences in Five-Factor scores were not statistically significant (*P* > 0.05). The analysis suggests that the cognitive-structural framework-based experiential education programme significantly improved two types of cognitive biases in schizophrenia patients with delusions: “safety behaviours” and “precipitate judgements”. Regarding deeper cognitive biases such as “insufficient mentalisation” and “belief rigidity bias”, although a downward trend was observed, it has not yet reached statistical significance. The intervention period in this study lasted four weeks. The observed decline in efficacy suggests that extending the intervention duration or combining it with individual cognitive behavioural therapy may further enhance therapeutic outcomes. Whether through traditional cognitive behavioural therapy or experiential education, improvements in cognitive biases exhibit characteristics of selectivity and phased progression. The marked improvement in safety behaviour is directly attributable to the “Choice of Response” module within the curriculum. In the scenario-based demonstration, patients gained an intuitive understanding that whilst “safety behaviours” such as suspicion, avoidance, and blame may temporarily alleviate anxiety, they ultimately lead to relationship breakdown and reinforce delusions. Conversely, proactive coping strategies like communication, verification, and perspective-taking—though requiring tolerance of short-term discomfort—yield superior interpersonal outcomes. This concrete visualisation of behavioural consequences effectively reduces patients’ reliance on “safe behaviours”. The marked improvement in rash conclusions stems from two core modules: “evidence collection training” and “multi-dimensional thinking training”. Through the gradual revelation of information in scenarios such as ‘a needle in the pillow’ and ‘colleagues ceasing conversation’, the patient repeatedly undergoes a cognitive conflict process of ‘initial misjudgement → insufficient evidence → emergence of truth’. This effectively curbs the cognitive habit of hasty conclusions. This approach of reshaping automated information processing patterns through structured, repetitive practice aligns with the principles of metacognitive training (34).This pattern of findings is highly consistent with the efficacy observations of cognitive behavioural therapy for patients with paranoid schizophrenia delusions by Wang Yiquan et al. (35), aligns with the research findings of Tim Bastiaens et al. that ‘compared to other subscales, safety behaviours demonstrate relatively better discriminative validity’ (36), and is also consistent with the research conclusion of Korkmaz et al. that ‘results are similar to previous findings’ (37).DACOBS scores showed a significant improvement in safety behaviour and hasty conclusions only; the remaining factors showed a trend towards improvement but did not reach statistical significance. This suggests that short-term interventions are more effective at addressing superficial cognitive biases, whilst deeper biases such as fixed beliefs and insufficient mentalisation require longer intervention periods or combined with individualised treatment. This pattern of selective improvement is consistent with previous studies on CBT-p and MCT, and is not unique to this intervention.

### 4.4 Changes in the P300 event-related potential: preliminary neurophysiological findings

The results of this study indicate that in the post-training observation group, the latency periods of N1, N2, and P3 were shorter than those in the control group, whilst the amplitudes of N1 and P3 were increased, with statistically significant differences (*P* < 0.05).Changes in the aforementioned indicators represent alterations at the neurophysiological level only and do not directly equate to an improvement in clinical cognitive function. Changes in the latency and amplitude of the P300 reflect alterations in neural information processing, rather than improvements in cognitive abilities such as attention, memory and executive function in real-world settings. As this study did not include concurrent neuropsychological testing, it is not possible to establish a direct causal link between the electrophysiological changes and the improvement in cognitive function. There were no significant differences in N2 amplitude between groups, which may be attributed to the limited sample size and insufficient statistical power; this may be related to the fact that the intervention had a stronger modulatory effect on late-stage cognitive processing (P3) than on early-stage automatic processing (N2).Scenario-based educational programmes employ diverse situational settings and task requirements to deliver comprehensive training across multiple cognitive domains, including attention, memory, and executive function, thereby promoting improvements in patients’ cognitive abilities. The programme requires patients to shift perspectives between different scenarios, retain multiple possibilities in memory, and make rational decisions. These tasks continuously challenge patients’ attention, working memory, and cognitive flexibility, constituting a targeted form of cognitive rehabilitation training. Extensive literature indicates that patients with schizophrenia exhibit significantly prolonged P300 latency and markedly reduced wave amplitude compared to healthy controls (38–40). Furthermore, first-degree relatives of schizophrenia patients without symptoms also demonstrate P300 abnormalities (41). In studies examining the correlation between P300 components and cognitive impairment, Francisco et al. (41) observed that reduced P300 amplitude in schizophrenia patients was associated with executive function deficits. Wang et al. (42) reported that diminished P300 amplitude in schizophrenia patients correlated with impairments in language processing and working memory. In summary, the P300 has achieved highly significant progress in research concerning the cognitive domain of schizophrenia patients and may serve as an electrophysiological indicator for assessing their cognitive function. The shortened P300 latency and increased amplitude observed in this study cannot rule out the influence of confounding factors such as the practice effect, medication stabilisation and the ward environment. Consequently, these findings can only be regarded as preliminary electrophysiological evidence related to the intervention and require further validation using standardised neurocognitive scales. Based on the current findings, this study cannot yet conclude that the intervention improved cognitive function.

## 5 Limitations and future prospects

This study has the following main limitations: (1)Inadequate control conditions: no attention-matching control group was established, making it impossible to distinguish between the specific and non-specific effects of the intervention. (2) This was a single-centre study that did not employ an intention-to-treat (ITT) analysis: all patients were recruited from the same hospital, limiting the study’s external validity; furthermore, this design may slightly overestimate the effect size, and therefore the results should be interpreted with caution. (3) The intervention period was relatively short and no follow-up was conducted: the intervention in this study lasted only four weeks, and no follow-up assessments were carried out at one, three or six months post-intervention, making it impossible to verify the sustainability of the intervention’s effects. (4) There are limitations to the interpretation of ERP results: standardised neuropsychological tests were not used in conjunction with the analysis, and the study itself remains subject to various confounding factors. (5) Lack of technological innovation: Emerging technologies such as virtual reality and artificial intelligence were not incorporated; the innovation of this study lies primarily in the low-cost and replicable nature of the intervention model. (6) The timing of the distribution of incentives differed between the two groups, which may have introduced bias.

Future research could be conducted in the following areas: (1)Introducing a control group with attention-matching; (2)Conducting multicentre, large-scale randomised controlled trials using intention-to-treat (ITT) analysis; (3)Extending the intervention period and incorporating long-term follow-up; (4)Concurrently administering standardised neuropsychological tests; (5)Further exploring a stepwise intervention model; (6)Standardising the timing of reward distribution in future studies.

## 6 Conclusion

This study demonstrates that scenario-based education grounded in the cognitive structure framework can, in the short term and selectively, improve coping strategies, delusional symptoms and certain cognitive distortions (safety-seeking behaviour and jumping to conclusions) in patients with schizophrenia accompanied by delusions, and is associated with preliminary changes in the ERP-P300.Due to limitations such as a single-centre design, the absence of a control group with matched baseline characteristics, and the lack of long-term follow-up, the conclusions of this study are exploratory in nature; the specificity, long-term effects and generalisability of the intervention still require validation through higher-quality studies.

## Data Availability

The raw data supporting the conclusions of this article will be made available by the authors, without undue reservation.
